# Study of Metabolite Detectability in Simultaneous Profiling of Amine/Phenol and Hydroxyl Submetabolomes by Analyzing a Mixture of Two Separately Dansyl-Labeled Samples

**DOI:** 10.3390/metabo15080496

**Published:** 2025-07-23

**Authors:** Sicheng Quan, Shuang Zhao, Liang Li

**Affiliations:** 1 The Metabolomics Innovation Centre, University of Alberta, Edmonton, AB T6G 2N4, Canada; quan@ualberta.ca (S.Q.); szhao1@ualberta.ca (S.Z.); 2 Department of Chemistry, University of Alberta, Edmonton, AB T6G 2G2, Canada

**Keywords:** chemical isotope labeling (CIL) LC-MS, comprehensive metabolome profiling, rapid sample processing, dansyl labeling

## Abstract

Background: Liquid chromatography-mass spectrometry (LC-MS), widely used in metabolomics, is often limited by low ionization efficiency and ion suppression, which reduce overall metabolite detectability and quantification accuracy. To address these challenges, chemical isotope labeling (CIL) LC-MS has emerged as a powerful approach, offering high sensitivity, accurate quantification, and broad metabolome coverage. This method enables comprehensive profiling by targeting multiple submetabolomes. Specifically, amine-/phenol- and hydroxyl-containing metabolites are labeled using dansyl chloride under distinct reaction conditions. While this strategy provides extensive coverage, the sequential analysis of each submetabolome reduces throughput. To overcome this limitation, we propose a two-channel mixing strategy to improve analytical efficiency. Methods: In this approach, samples labeled separately for the amine/phenol and hydroxyl submetabolomes are combined prior to LC-MS analysis, leveraging the common use of dansyl chloride as the labeling reagent. This integration effectively doubles throughput by reducing LC-MS runtime and associated costs. The method was evaluated using human urine and serum samples, focusing on peak pair detectability and metabolite identification. A proof-of-concept study was also conducted to assess the approach’s applicability in putative biomarker discovery. Results: Results demonstrate that the two-channel mixing strategy enhances throughput while maintaining analytical robustness. Conclusions: This method is particularly suitable for large-scale studies that require rapid sample processing, where high efficiency is essential.

## 1. Introduction

Liquid chromatography-mass spectrometry (LC-MS) is one of the most widely used platforms for metabolome analysis due to its broad metabolite coverage. However, metabolite detectability is often reduced by the low ionization efficiency of many metabolites and the strong ion suppression of co-eluting metabolites. To address these challenges, various analytical methods have been developed to improve ionization and simplify the sample matrix by targeting different classes of metabolites. For example, reverse-phase liquid chromatography (RPLC) and hydrophilic interaction liquid chromatography (HILIC) are often coupled with positive or negative ion modes in mass spectrometry to analyze metabolites with varying polarities and hydrophobicities [1,2,3,4,5,6]. Despite these advancements, metabolome coverage in LC-MS-based metabolomics remains limited. Chemical isotope labeling (CIL) LC-MS has emerged as a powerful solution to enhance detection sensitivity as well as improve metabolite quantification accuracy [7,8]. By chemically labeling metabolites with optimized reagents prior to LC-MS analysis, this technique significantly increases signal-to-noise ratios, thereby enhancing metabolite detection [9,10]. Moreover, if many co-eluting metabolites are ionized efficiently, strong ion suppression among these ions may reduce detectability. Therefore, it is essential to examine the interplay between separation efficiency and ion suppression in order to achieve optimal sample throughput and metabolite coverage.

Our group previously developed a four-channel high-performance CIL LC-MS technique that comprehensively profiles the metabolome by targeting four chemical classes of metabolites (submetabolomes): amine-/phenol-, hydroxyl-, carbonyl-, and carboxyl-containing metabolites [11]. Specific and optimized labeling reactions were applied for each class: dansyl chloride (DnsCl) for amine-/phenol-containing [12] and hydroxyl-containing metabolites [13] under distinct chemical reaction conditions, p-dimethylaminophenacyl (DmPA) bromide for carboxyl-containing metabolites [14], and dansylhydrazine (DnsHz) for carbonyl-containing metabolites [15]. These labeling strategies increase metabolite hydrophobicity and ionization efficiency, thereby enhancing retention in reversed-phase (RP) LC, even for very polar and ionic metabolites, and increasing signal intensity in MS [16]. As a result, this approach achieves extensive metabolome coverage, enabling the intricate profiling of complex biological samples.

Similar to conventional LC-MS methods where samples are analyzed in multiple runs (e.g., four LC runs using a combination of RPLC and hydrophilic interaction LC with positive and negative ion modes), the four-channel CIL LC-MS technique requires sequential analysis of each submetabolome. Consequently, a single sample may require four LC-MS runs; one example was to perform each run with a runtime of approximately 20 min [17], totaling 80 min per sample. This extended analysis time may not be desirable for large-scale studies, such as population-based metabolomics, where thousands of samples must be processed under time constraints. In such cases, speed and efficiency may take precedence over comprehensive metabolite coverage. While reducing analysis time may result in fewer detected metabolites, it remains a practical trade-off for high-throughput screening and time-sensitive studies [18].

In this study, we report a two-channel mixing strategy that combines amine/phenol submetabolomes and hydroxyl-submetabolomes after labeling but prior to LC-MS analysis. Since both submetabolomes utilize DnsCl as the labeling reagent, this approach enables the simultaneous detection of amine-, phenol-, and hydroxyl-containing metabolites in a single LC-MS run. This combination has the potential to significantly reduce analysis time while maintaining essential metabolite coverage. In this study, human urine and serum samples were analyzed using the two-channel mixing strategy to evaluate its performance in metabolome profiling, including peak detectability and metabolite identification. A proof-of-concept study was further conducted to assess its practicality for putative biomarker discovery. Our results demonstrate that while this approach sacrifices some metabolome coverage, it significantly enhances throughput, making it particularly suitable for large-scale and time-sensitive studies where efficient sample analysis is critical.

## 2. Materials and Methods

**Figure 1:**
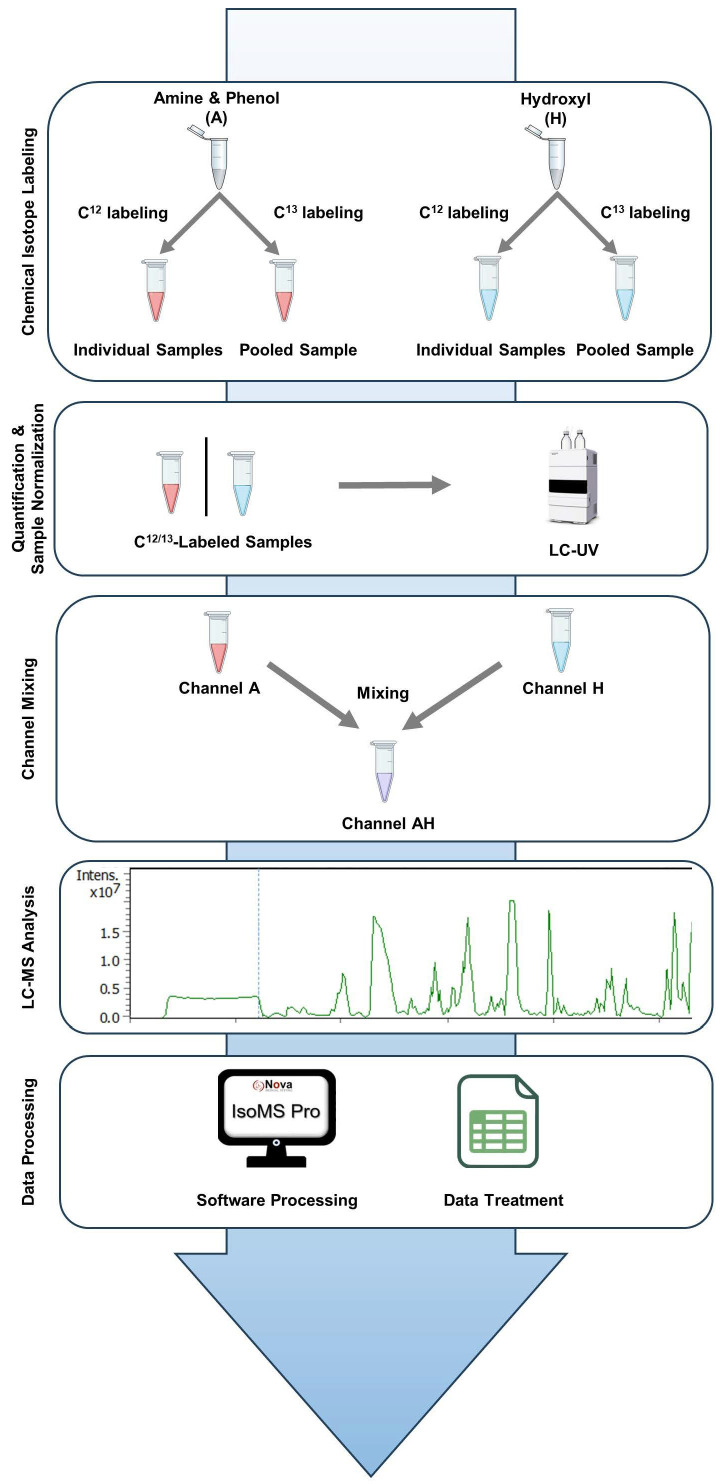
Workflow of the two-channel mixing-based CIL LC/MS analysis.

**Study Workflow.** The study workflow consists of five key steps, as illustrated in Figure 1. Step 1 is chemical isotope labeling. Metabolites containing amine/phenol (A) and hydroxyl (H) groups were chemically tagged to enhance detection and quantitative measurement. Individual samples were labeled with ^12^C-Dansyl Chloride (^12^C-DnsCl) (light labeling). A pooled sample, created by combining equal aliquots of all individual samples, was labeled with ^13^C-Dansyl Chloride (^13^C-DnsCl) (heavy labeling). The heavy-labeled pooled sample served as an internal standard for all light-labeled samples, with equal amounts added to each sample based on quantification results (see below), forming a ^12^C/^13^C mixture for LC-MS analysis. Step 2 involves sample quantification and normalization. Metabolome concentrations were measured using LC-UV and normalized to ensure uniformity for subsequent dilution and mixing. Step 3 is channel mixing. Equal parts of the amine/phenol (A) and hydroxyl (H) labeled samples were combined to create an AH mixture (AH) for combined analysis. Step 4 is LC-MS analysis. The A-, H-, and AH-labeled samples were analyzed by LC-MS. Metabolites were detected as peak pairs, distinguishing true metabolites from noise or unlabeled background signals. Step 5 is data processing. Raw LC-MS data were processed using IsoMS Pro (V1.4.0) software for peak pair detection, quantification, and metabolite identification. Further statistical analysis was performed in IsoMS Pro and Microsoft Excel (2016).

To evaluate the method’s performance, 16 urine samples and 15 serum samples were analyzed across individual channels (A and H) and the mixed channel (AH). A proof-of-concept study was conducted using 16 urine samples from two donors to further validate the approach’s feasibility for putative biomarker discovery.

**Chemicals and Reagents.** All chemicals and reagents, unless otherwise specified, were obtained from Sigma-Aldrich Canada (St. Louis, MO, USA). Chemical isotope labeling kits (CIL-4101-KT and CIL-4145-KT) were purchased from Nova Medical Testing Inc. (Edmonton, AB, Canada, www.novamt.com, accessed on 3 October 2022). LC-MS-grade solvents, including water, acetonitrile (ACN), and methanol (MeOH), were procured from Thermo Fisher Scientific (Waltham, MA, USA) and Honeywell Research Chemicals (Charlotte, NC, USA).

**Sample Pretreatment.** Serum samples (30 μL) were mixed with 90 μL of pre-cooled methanol (MeOH), vortexed, and centrifuged at 10,000× *g* for 10 min to remove proteins. The supernatant was frozen at −20 °C for 1 h, re-centrifuged, and vacuum-dried. The residue was reconstituted with 25 μL of H_2_O for amine/phenol labeling or 25 μL of 3:1 ACN/H_2_O for hydroxyl labeling. Pre-filtered urine samples (12.5 μL) were thawed at 4 °C and centrifuged at 10,000× *g* for 10 min. For amine/phenol labeling, samples were diluted 4× with 37.5 μL of H_2_O. For hydroxyl labeling, samples were diluted with 37.5 μL of ACN to achieve a 3:1 ACN/H_2_O matrix. A 25 μL aliquot of each diluted sample was used for labeling.

**Chemical Isotope Labeling.** Chemical isotope labeling was performed following standard procedures provided in the CIL-4101-KT and CIL-4145-KT kits (Nova Medical Testing Inc., Edmonton, AB, Canada). Amine-/phenol-containing metabolites were labeled through a dansylation reaction, while hydroxyl-containing metabolites were labeled using a base-activated dansylation reaction.

**Sample Normalization**. Metabolite concentrations were quantified using a validated LC-UV method on an Agilent 1220 Infinity II HPLC system with a variable wavelength detector (VWD) [19]. Mobile phase A was 0.1% formic acid in 5% ACN/H_2_O, and mobile phase B was 0.1% formic acid in ACN. The LC gradient was set as follows: 0 min, 0% B; 0.01 min, 95% B; 2.5 min, 95% B; 3 min, 0% B; 6 min, 0% B. The flow rate was 0.45 mL/min. Metabolites were detected by measuring UV absorption at 338 nm. Metabolite concentrations were determined from the area under the curve, and equal amounts of ^12^C- and ^13^C-labeled samples were mixed for subsequent LC-MS analysis.

**LC-MS Analysis.** Labeled urine samples were analyzed using a Dionex UltiMate 3000 UHPLC system (Thermo Fisher Scientific, USA) coupled to a Maxis II QTOF mass spectrometer (Bruker Daltonics, Billerica, MA, USA) with an Eclipse Plus C18 column (2.1 × 100 mm, 1.8 μm, 95 Å, Agilent, Santa Clara, CA, USA). Mobile phase A was 0.1% formic acid in water, and mobile phase B was 0.1% formic acid in ACN. The LC gradient was set as follows: 0 min, 25% B; 10 min, 99% B; 15 min, 99% B; 15.1 min, 25% B; 18 min, 25% B. The flow rate was 400 µL/min, with a column temperature at 40 °C. MS data were acquired in positive ion mode at 1 Hz.

Similarly, serum samples were analyzed using an Agilent 1290 Infinities II LC system coupled with an Impact II QTOF mass spectrometer (Bruker Daltonics, Billerica, MA, USA), under identical conditions as for urine samples.

**Data Processing and Metabolite Identification.** Raw LC-MS data were processed using IsoMS Pro software, including peak pair detection, quantification, filtering, alignment, and imputation [20]. Metabolite identification followed a three-tiered approach using the NovaMT Metabolite Databases 2.0 (Nova Medical Testing Inc., Canada). In tier 1, peak pairs matched against the labeled metabolite library (CIL Library) based on accurate mass and retention time as positive identification. In tier 2, remaining peak pairs matched to the linked identity (LI) library, containing 9000+ pathway-related metabolites with predicted retention times, generating high-confidence putative identifications. In tier 3, remaining peak pairs matched to the MyCompoundID (MCID) Library (www.mycompoundid.org, accessed on 16 April 2024), which includes 8021 known endogenous metabolites in the zero-reaction library, 375,809 predicted metabolites in the one-reaction library, and 10,583,901 predicted metabolites in the two-reaction library. The resulting metabolite-intensity table was used for further statistical analysis.

**Peak Pair Analysis.** Peak pair detection was performed using IsoMS Pro, which identifies labeled metabolites through the detection of co-eluting ^12^C/^13^C-labeled ion pairs (i.e., peak pairs) that exhibit the expected mass difference of 2.0067 Da. Overlapping and unique peak pairs were determined by comparing *m*/*z* and retention time across different channels. Peak pairs were considered overlapping if both *m*/*z* and retention time matched within a defined tolerance window. All comparisons were performed after data alignment and filtering. Post-channel mixing peak pair loss was assessed by comparing LC-MS spectra between individual channels (A or H) and the AH mixture at the same retention time and *m*/*z* region. First, a peak pair that was clearly detected in the individual channel analysis and matched the expected isotopic spacing of 2.0067 Da between ^12^C/^13^C-labeled peak pairs was selected. The relevant *m*/*z* window was then enlarged to enhance clarity of peak shape, spacing, and intensity. The same retention time and *m*/*z* region were examined in the AH mixture. A peak pair was considered lost if absent or below the detection threshold in the AH mixture, but clearly present in the single channel.

**Statistical Analysis.** Metabolite-intensity data were analyzed using IsoMS Pro for univariate and multivariate analyses. Significant metabolites were determined using volcano plots with the following criteria: fold change > 1.2 or <0.83, *p*-value < 0.05, and q-value (FDR-adjusted *p*-value) < 0.25. All three criteria were used to define significant metabolites in volcano plot analyses. The relaxed q-value threshold served to reduce the risk of false negatives and avoid overlooking potentially meaningful metabolites, while the application of fold-change and *p*-value criteria further refined the selection of significant features. Additional analyses, such as Venn diagrams, were performed using Microsoft Excel.

## 3. Results and Discussion

**Figure 2:**
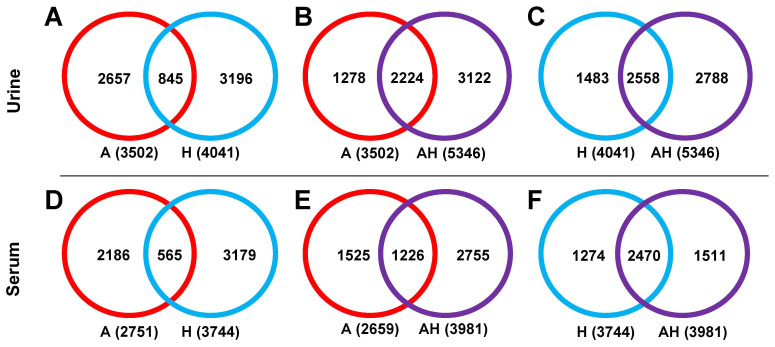
Venn diagrams illustrating the number of detected peak pairs in the A channel (red), H channel (blue), and AH mixture (purple) for urine (**A**–**C**) and serum (**D**–**F**) samples. In each channel, a labeled metabolite is detected as a co-eluting ^12^C/^13^C-labeled ion pair (i.e., peak pair) that exhibits the expected mass difference of 2.0067 Da. Overlapping and unique peak pairs were determined by comparing *m*/*z* and retention time across different channels. A peak pair was considered overlapping if both *m*/*z* and retention time matched within a defined tolerance window.

**Peak Pair Detectability.** Peak pair detection in the single-channel analysis of amine/phenol submetabolome (A) and hydroxyl submetabolome (H) is summarized in Figure 2 for urine (Figure 2A–C) and serum samples (Figure 2D–F). Further information is provided in Supplementary Tables S1 and S2. In urine samples, 3502 peak pairs were detected in the A channel and 4041 were detected in the H channel (Figure 2A). Less than 25.0% of the detected peak pairs overlapped between the two channels, highlighting the specificity of the chemical labeling techniques employed and the need for a four-channel labeling strategy to achieve comprehensive metabolite coverage.

The AH mixture captured 2224 peak pairs from the A channel (63.5% coverage, Figure 2B) and 2558 peak pairs (63.3% coverage, Figure 2C) from the H channel. Similar results were observed for serum samples: 2751 peak pairs were detected in the A channel and 3744 in the H channel, with 80% of peak pairs being unique to each channel (Figure 2D). The AH mixture retained 1226 peak pairs from the A channel (46.1% coverage, Figure 2E) and 2470 from the H channel (66.0% coverage, Figure 2F).

Notably, in urine samples, the AH mixture captured a higher percentage of peak pairs from the A channel compared to the H channel, whereas in serum samples, the opposite trend was observed. This observation suggests differences in ion suppression effects between urine and serum, emphasizing that ion suppression varies with sample type. These findings indicate the importance of understanding ion suppression effects in metabolomic analyses to ensure accurate and comprehensive metabolite detection across different sample types.

**Figure 3:**
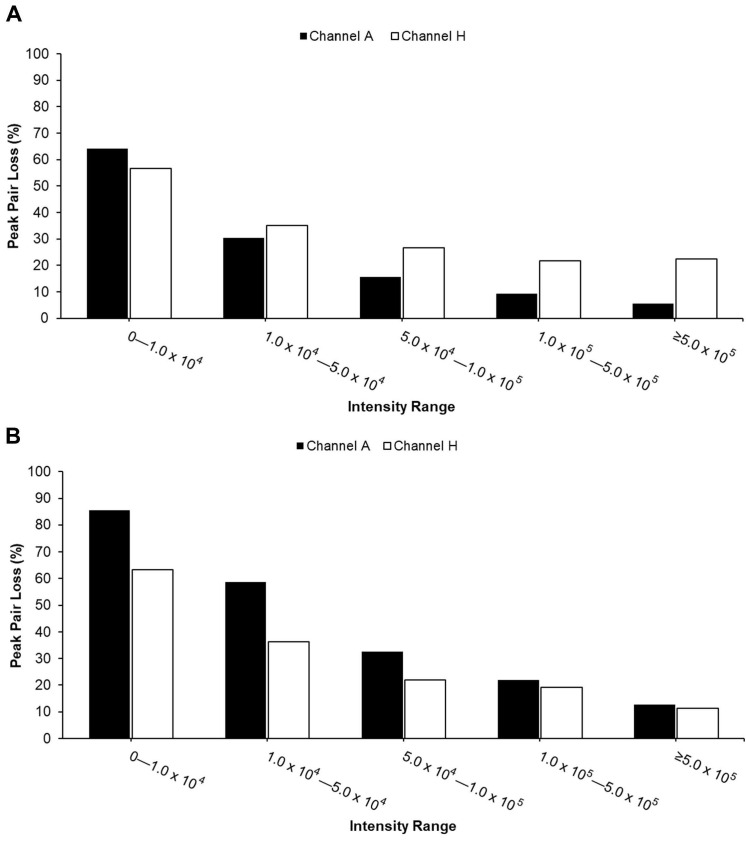
Percentage of peak pair loss in AH mixture across five intensity regions (0 to >500,000) for urine (**A**) and serum (**B**) samples. Peak pair loss is defined as peak pairs detected in individual A channels or H but absent in the AH mixture after sample mixing.

**Ion Suppression Effect.** To further investigate ion suppression, we calculated the percentage of peak pair loss in the AH channel, as illustrated in Figure 3. Peak pair loss was defined as follows: Peak pair loss = (Number of peak pairs detected in single channel but absent in AH channel)/(Number of peak pairs in single channel). Higher values indicate greater loss of peak pairs during channel mixing. The results showed that peak pairs at lower intensities were more susceptible to loss due to ion suppression across both sample types.

For urine samples, peak pair losses of the A channel were lower compared to the H channel in most of the intensity ranges, except for the lowest range (Figure 3A), suggesting that amine-/phenol-containing metabolites were better preserved during mixing. In contrast, serum samples displayed higher peak pair loss in the A channel compared to the H channel (Figure 3B), indicating a distinct detection preference depending on sample type and intensity region.

**Figure 4:**
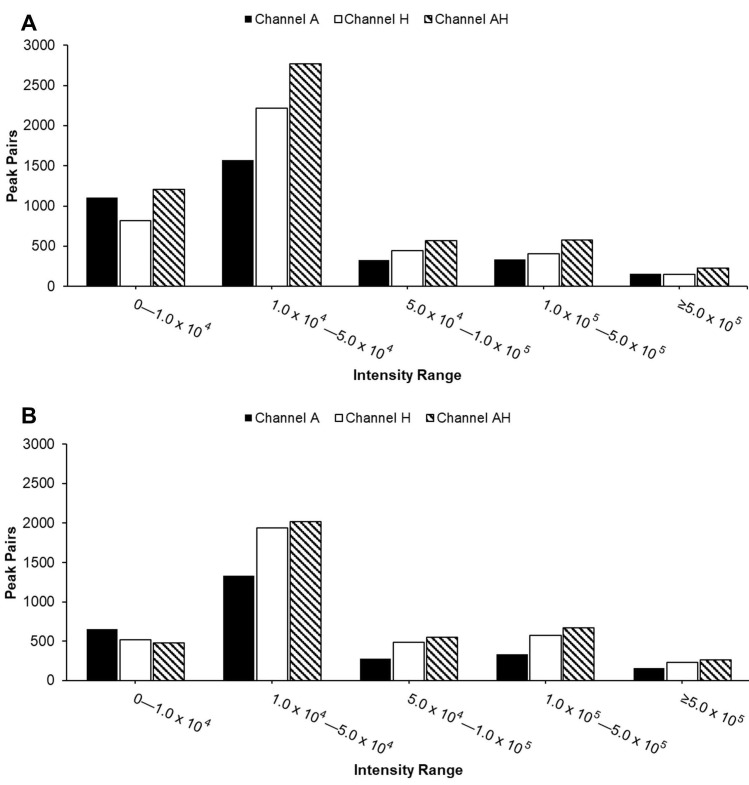
Distribution of peak pairs detected in the A channel, H channel, and AH mixture across five intensity ranges (from 0 to >500,000) for urine (**A**) and serum (**B**) samples. Each peak pair represents a labeled metabolite, detected as a co-eluting ^12^C/^13^C-labeled ion pair.

Compared to individual channels, the AH mixture consistently detected the highest number of peak pairs across nearly all intensity ranges (Figure 4), as expected due to the combination of metabolites from both A and H channels. Most peak pairs in both urine and serum samples were detected in the two lowest intensity ranges. The reduced peak pair coverage observed in Figure 2 can be attributed to significant ion suppression effects seen in Figure 3. While the overall distribution of peak pairs was similar between sample types, urine samples exhibited a higher abundance of peak pairs in the lowest intensity range, reflecting their intrinsic chemical complexity.

**Figure 5:**
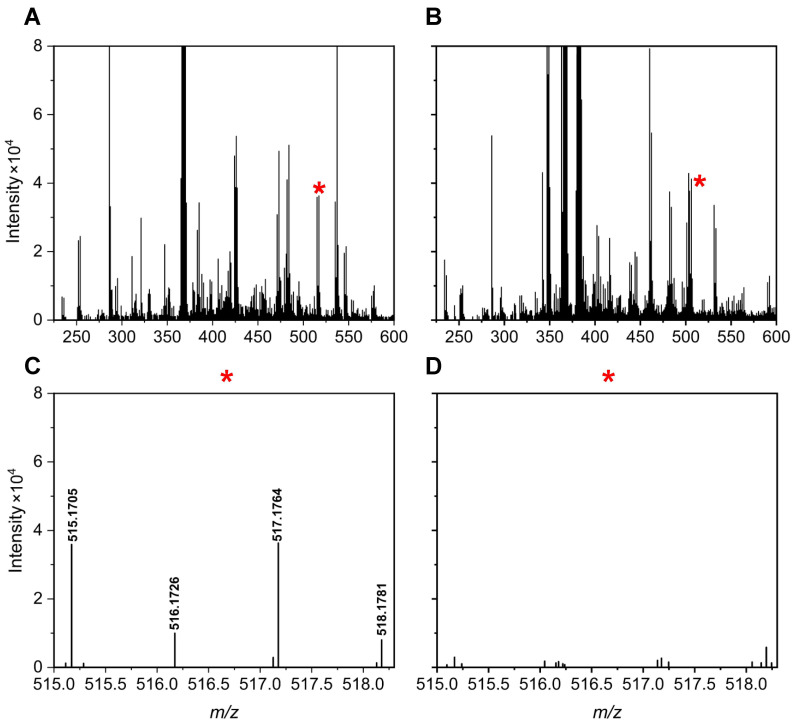
Mass spectra of a urine sample at 2.01 min for the A channel (**A**) and AH mixture (**B**). A region with peak pair loss is marked by a red * and enlarged for clarity: A channel enhanced (**C**) and AH mixture enhanced (**D**).

**Figure 6:**
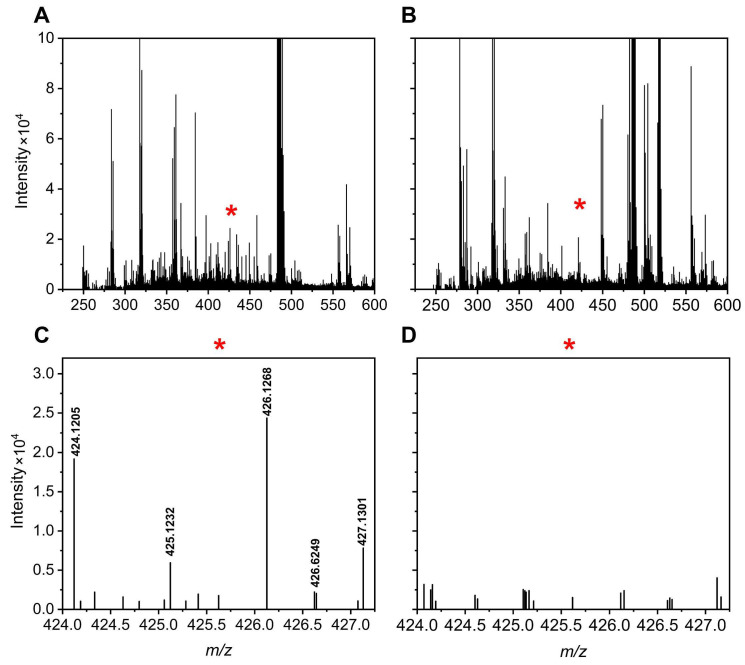
Mass spectra of a urine sample at 8.01 min for the H channel (**A**) and AH mixture (**B**). A region with peak pair loss is marked by a red * and enlarged for clarity: H channel enhanced (**C**) and AH mixture enhanced (**D**).

Figure 5 and Figure 6 illustrate specific examples of peak pairs lost post-mixing. For instance, a peak pair with an *m*/*z* value of 515.1705/517.1764, initially detected in channel A, disappeared in the mixed AH channel (Figure 5). Similarly, a peak pair with an *m*/*z* value of 424.1205/426.1268 found in channel H was absent after mixing (Figure 6).

One potential strategy to address ion suppression, particularly for low-intensity metabolites, is to incorporate sample fractionation during the preparation steps. This process separates complex samples into simpler fractions, thereby reducing matrix effects and enhancing detection sensitivity [21]. Additionally, LC separation conditions can be further optimized to match the composition of the two-channel mixture, as improved chromatographic resolution allows for better separation of metabolites, aiding in ionization efficiency. Alternatively, if comprehensive profiling is a priority, the original four-channel labeling strategy may be more appropriate. CIL significantly enhances the signal intensity of labeled metabolites, improving detectability, particularly for low-abundance compounds [22]. Overall, ion suppression is mainly caused by the inherent complexity of samples like urine and serum. There is an unavoidable trade-off between throughput and sensitivity, but channel mixing is customized for high-throughput analysis while accepting a minor reduction in metabolome coverage.

**Figure 7:**
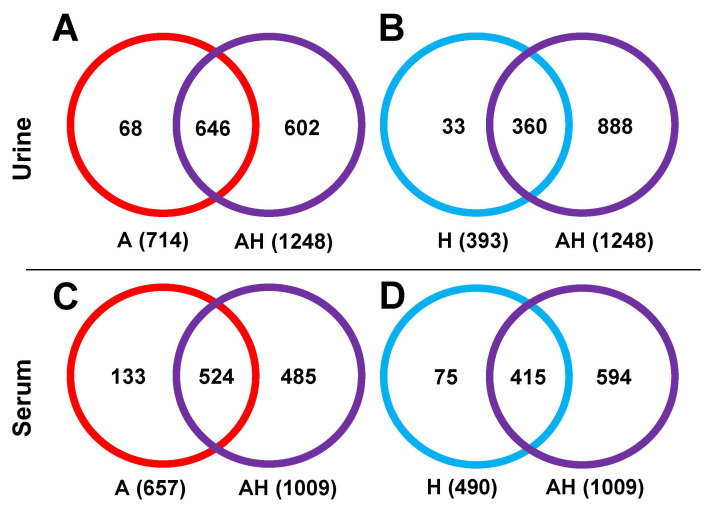
Venn diagram showing the number of identified metabolites in the A channel (red), H channel (blue), and AH mixture (purple) for urine (**A**,**B**) and serum (**C**,**D**) samples.

**Metabolite Identification.** The number of highly confident identified metabolites (tier 1 and tier 2) between single channels (A and H) and the AH mixture were compared, as shown in Figure 7. For urine samples, the A channel identified 714 metabolites, comprising 245 tier 1 and 469 tier 2 metabolites (Figure 7A). The H channel identified 393 metabolites, including 67 tier 1 and 326 tier 2 metabolites (Figure 7B). The AH combined channel covered 90.5% of the metabolites identified in the A channel and 91.6% of those identified in the H channel, indicating efficient retention of key metabolites. Similar results were observed for serum samples. Of the 657 metabolites identified in the A channel (325 tier 1 and 332 tier 2, Figure 7C), the AH combined channel captured 524 metabolites (79.8% coverage). For the H channel, 415 out of 490 metabolites (84.7%) were retained in the AH mixture (Figure 7D).

The higher coverage percentage of identified metabolites compared to the detected peak pairs suggests that, although some metabolite candidates may be lost in the combined channel analysis, the most critical information, i.e., high-confidence identified metabolites, is largely preserved. This highlights the robustness of the AH combined channel for metabolite identification, ensuring effective data integration without significant loss of essential findings.

The observed loss of coverage in the AH combined channel reflects the trade-off between analytical throughput and metabolite coverage; while some reduction in coverage was noted, a significantly higher throughput was achieved while maintaining an acceptable level of metabolite detection. Additionally, as shown in Figure 3, the coverage loss primarily occurred among peak pairs with low signal intensity, which are features that typically exhibit lower detection consistency and quantification accuracy [3].

**Proof of Concept Study.** To evaluate the feasibility of our method, we analyzed 16 urine samples from two distinct donors. Each donor contributed eight samples collected over three days at morning, noon, and evening intervals. These donors represented two unique population groups, providing valuable insights into the method’s practicality for real-world research, such as putative biomarker discovery.

**Figure 8:**
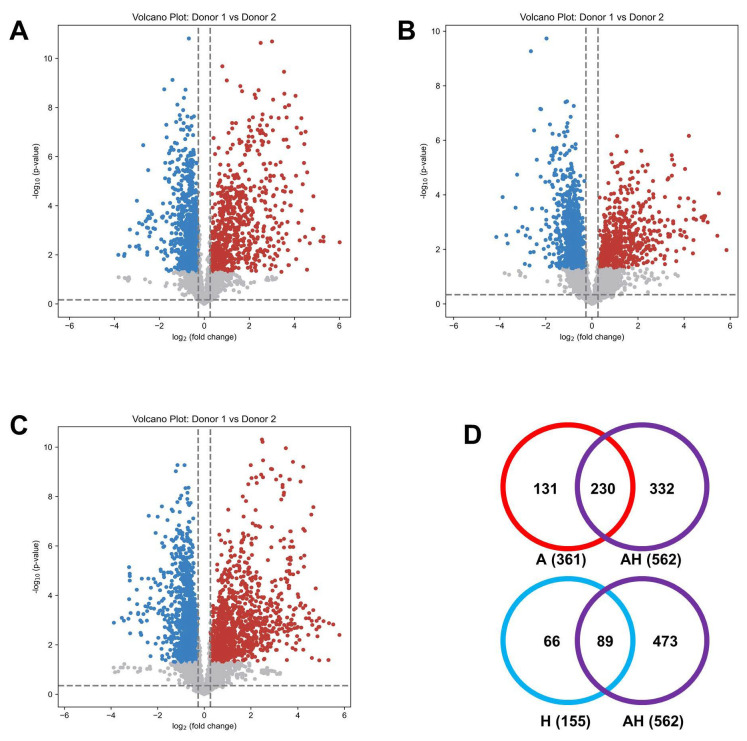
Volcano plots of binary comparisons of urine samples from two independent donors (Donor 1, Blue ; Donor 2, Red) for the A channel (**A**), H channel (**B**), and AH mixture (**C**). Venn diagram (**D**) illustrating the overlap of significantly changed metabolites (tier 1 and tier 2 only) among the A (red), H (blue), and AH (purple) channels.

Using volcano plots as a univariate analysis tool, we identified metabolites with significant differences, serving as potential biomarker candidates to differentiate samples between the two donors. Across channels A, H, and AH, we identified 1630, 1512, and 2569 significant metabolites, respectively (Figure 8A–C). A Venn diagram of highly confident identified metabolites (tier 1 and tier 2) (Figure 8D) revealed that the AH channel captured 63.7% and 57.4% of the significantly altered metabolites identified in the A and H channels, respectively. Despite detecting fewer metabolites overall compared to the combined totals of the A and H channels, the AH channel identified a substantial number of significant metabolites while reducing analysis time by half. Further information is provided in Supplemental Tables S3–S5.

**Figure 9:**
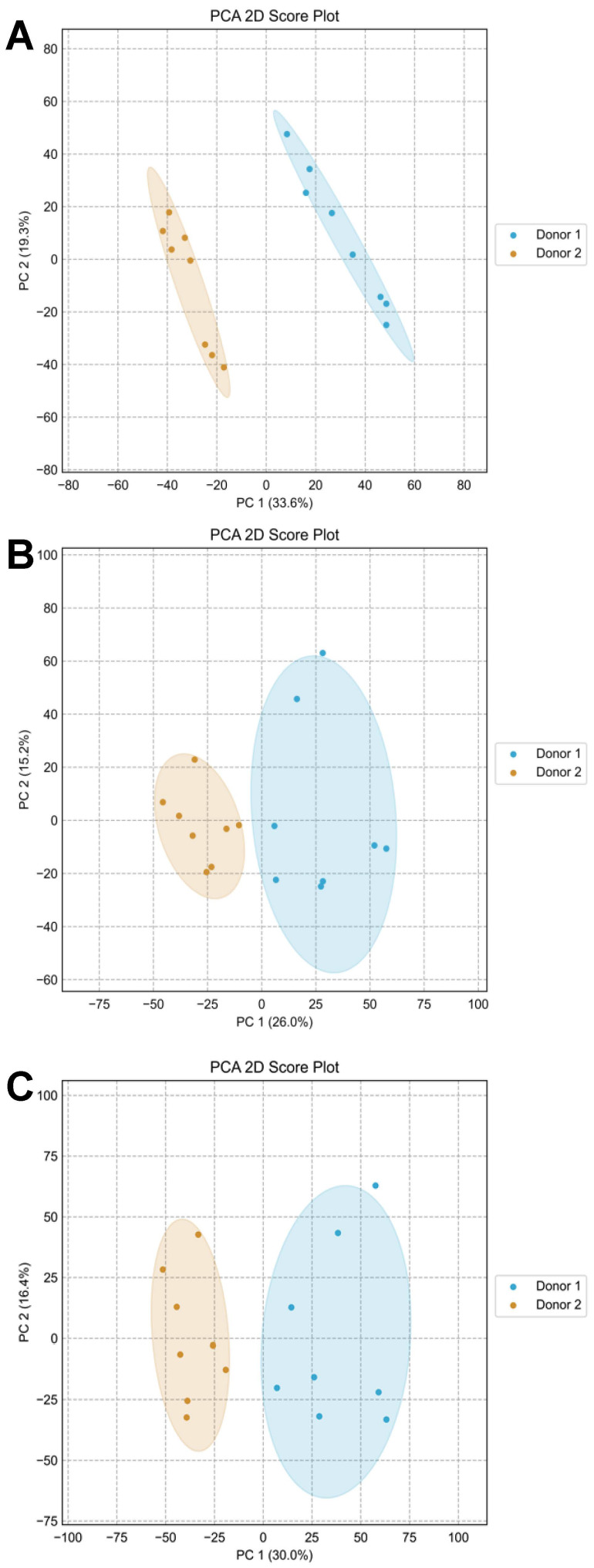
Principal component analysis of urine samples from two independent donors for the A channel (**A**), H channel (**B**), and AH mixture (**C**).

Principal Component Analysis (PCA) was conducted to visually examine the distribution of the 16 samples. The PCA for amine-/phenol-containing metabolites showed clear separation between the two donors, indicating distinct metabolite compositions (Figure 9A). In contrast, the PCA for hydroxyl-containing metabolites displayed slightly overlapping clusters, suggesting limited compositional differences for this metabolite subset (Figure 9B). Notably, the PCA for the AH mixture effectively integrated the unique characteristics from both A and H channels, resulting in clear separation and demonstrating successful data integration from both submetabolomes (Figure 9C).

Importantly, the eight samples collected from each donor at different times clustered closely together, reflecting the consistency and stability of each donor’s unique metabolite composition. This underscores the reliability of the CIL approach in preserving sample integrity over time. Additionally, despite the modest loss in coverage, both univariate and multivariate analyses demonstrate that the AH channel is still capable of capturing key metabolic differences between sample groups. This indicates that the loss of metabolite coverage through channel mixing does not significantly compromise the interpretability or validity of results. Moreover, the method’s ability to accurately capture an individual’s metabolic fingerprint across multiple sampling instances highlights its potential for biomarker exploration and longitudinal studies.

**Future Directions.** The two-channel mixing method designed was created to preserve group-wise comparability while improving throughput, rather than to optimize for absolute sensitivity or dynamic range. As a result, our study was focused on relative quantification across biological samples, not absolute quantification. We propose standard-based validation for evaluating sensitivity, linearity, and dynamic range to be conducted as a future direction [23].

The proposed two-channel mixing method reduces LC-MS runtime by half, as only one injection is needed due to the pre-analysis combination of amine-/phenol-labeled and hydroxyl-labeled samples. This reduction in time required per sample allows for substantial improvements in throughput that would positively impact studies in which thousands of samples must be processed, such as population-scale or longitudinal studies [24]. Additionally, the reduced runtime minimizes instrument downtime and required maintenance, allowing for continuous large-scale sample acquisition. Channel mixing after labeling the samples creates a modular workflow, supporting future automation [25]. This modular workflow allows our approach to be compatible with standardized, parallel sample preparation workflows, resulting in increased scalability and reproducibility across large datasets.

In this study, the two-channel approach was applied to human urine and serum samples, two biologically distinct matrices that differ in protein content, salt load, and metabolite complexity [26]. The results showed that the majority of high-confidence metabolites identified in individual channels were retained after mixing (>90% retained in AH mixture from urine A and H channels; >80% retained in AH mixture from serum A and H channels). Due to these results, we anticipate that this method can be applied to other complex sample types such as saliva, tissue, or cell lysate with only minor protocol adjustments.

To address current limitations related to sample types and metabolite coverage, future studies will focus on extending the two-channel mixing strategy to more complex biological matrices, such as tissues and cultured cells, which often require additional pretreatment steps [27]. Evaluating the method’s performance across diverse sample types will help establish its robustness and generalizability. Additionally, while the two-channel approach captures a broad range of metabolomic features, specific one-channel analyses or targeted validation workflows may be employed in parallel to confirm key findings or to provide enhanced coverage of certain metabolite classes that may be underrepresented. These combined strategies will help to further improve the analytical depth and applicability of the method in both exploratory and hypothesis-driven studies.

## 4. Conclusions

This study proposed a two-channel mixing approach based on the previously reported four-channel CIL LC-MS technique. In this method, samples labeled for the amine/phenol submetabolome and hydroxyl submetabolome were combined into a single channel for LC-MS analysis, effectively reducing the LC-MS running time by half by requiring only one injection instead of two separate ones. The performance of the two-channel approach was evaluated based on peak pair detection, ion suppression effects, and metabolite identification. Although the number of detected metabolites (peak pairs) decreased compared to individual LC-MS analyses of each submetabolome, the reduction was found to be acceptable. More importantly, the method retained most of the high-confidence identified metabolites, demonstrating its reliability in preserving critical metabolic information. A proof-of-concept study validated the practicality of this approach in real-world applications. This approach offers several advantages that are particularly relevant for clinical and large-scale studies. The channel mixing method effectively halves instrument runtime, enabling high-throughput analysis in settings where large sample volumes must be processed efficiently, such as clinical laboratories and large cohort studies. High-throughput analysis has recently established its critical role in the clinical setting, with the use of rapid next-generation sequencing during the SARS-CoV-2 pandemic to acquire retrospective data from large numbers of samples needing immediate attention [28]. Additionally, decreased instrument runtime decreases overall costs due to the reduction in solvent consumption, instrument wear, and personnel time. Importantly, this method boasts a simplified workflow while retaining the majority of high-confidence metabolites, striking a practical balance between analytical depth and operational feasibility and supporting greater scalability and analytical consistency. This method appears to be especially beneficial for longitudinal studies and biobank-scale analyses, where large numbers of samples are stored for long periods of time. A simplified workflow may improve reproducibility and aid in decreasing differences seen within an individual sample [29]. Additionally, the concurrent profiling of both the amine/phenol and hydroxyl submetabolomes supports metabolic network modeling, multi-omics integration, and other systems biology applications conducted in population-level studies. This study demonstrates that, despite some loss of coverage, the two-channel mixing strategy is highly advantageous for high-throughput environments requiring rapid sample processing. Its time efficiency and ability to retain key metabolite information make it a valuable tool for large-scale metabolomics studies and other applications demanding efficient analysis.

## Data Availability

All datasets are available upon request from the authors.

## Supplementary Materials

The following supporting information can be downloaded at: https://www.mdpi.com/article/10.3390/metabo15080496/s1, Supplemental Tables S1 and S2: Peak pair detection results for urine and serum samples, respectively. Supplemental Tables S3–S5: Significant metabolites identified from volcano plot analyses comparing samples from donor 1 and donor 2, corresponding to Channel A, Channel H, and Channel AH, respectively.
